# Optimizing Order Sets With a Large Language Model–Powered Multiagent System

**DOI:** 10.1001/jamanetworkopen.2025.33277

**Published:** 2025-09-23

**Authors:** Siru Liu, Sean S. Huang, Allison B. McCoy, Aileen P. Wright, Sara Horst, Adam Wright

**Affiliations:** 1 Department of Biomedical Informatics, Vanderbilt University Medical Center, Nashville, Tennessee; 2 Department of Computer Science, Vanderbilt University, Nashville, Tennessee; 3 Department of Medicine, Vanderbilt University Medical Center, Nashville, Tennessee

## Abstract

**Importance:**

Optimizing order sets is vital to enhance clinical decision support and improve patient care. Manual review is resource intensive and cannot timely identify potential improvements in order sets.

**Objective:**

To develop and evaluate the utility of a large language model (LLM)–powered multiagent system in optimizing order sets.

**Design, Setting, and Participants:**

A multiagent system was developed and evaluated between January 1, 2024, and December 31, 2024, which comprised agents for content critique, dynamic search, knowledge retrieval, medication verification, and suggestion summarization. A filter was developed to align suggestion usefulness scores with expert preferences. Experiment 1 evaluated 735 generated suggestions from a multiagent system developed for optimizing order sets, which were assessed by 3 physicians for 9 order sets and by 1 physician for 62 order sets. Experiment 2 implemented an LLM-as-a-judge approach to align generated suggestions with expert ratings and developed a filter to further refine the system’s performance. The study was performed at Vanderbilt University Medical Center. A total of 735 suggestions for 71 order sets at VUMC were evaluated by 3 physicians.

**Main Outcomes and Measures:**

The ratings of accuracy, usefulness, feasibility, and impact; interrater agreement; and alignment against historical ordering data.

**Results:**

In evaluation 1 of experiment 1, the median values for the number of suggestions scoring 4 or higher at the order set level were 5 (IQR, 5-6) for the metrics of accuracy, 2 (IQR, 1-4) for usefulness, 1 (IQR, 0-3) for feasibility, and 1 (IQR, 0-2) for impact. Of 96 suggestions, 44 (46%; 95% CI, 36%-56%) aligned with historical ordering patterns. In evaluation 2 of experiment 1, 639 suggestions were generated for 62 order sets; 52 order sets had at least 1 useful suggestion, with a median of 2 (IQR, 1-3) useful suggestions. Overall, 122 suggestions (19%; 95% CI, 16%-22%) were rated as useful. After expert alignment, Cohen κ improved from 0.06 to 0.41. Filtering using the aligned scores reduced total suggestions by 29% while retaining 92% of useful suggestions.

**Conclusions and Relevance:**

In this cohort study of an LLM-powered multiagent system for optimizing order sets, leveraging LLMs and multiagent systems provided a scalable approach. Alignment with a small set of expert ratings significantly enhanced the LLM evaluation. Future research could refine reasoning capabilities and integrate useful suggestions into electronic health records, while engaging end-users as artificial intelligence–supported reviewers.

## Key Points

**Question:**

What is the utility of a large language model–powered multiagent system in generating suggestions to optimize order sets compared with expert evaluation?

**Findings:**

In this cohort study including 735 suggestions for 71 order sets, 96 suggestions for 9 order sets were evaluated by 3 physicians, and 639 suggestions for 62 order sets were evaluated by 1 physician. The median number of useful suggestions per order set was 2 in both evaluations. Among the 96 suggestions, 54% were rated highly accurate (score ≥4), while 19% were rated highly useful, 16% feasible, and 12% as having a direct impact.

**Meaning:**

Results of this study suggest that multiagent systems offer a scalable and effective approach to enhancing order set optimization.

## Teaser

This study evaluates a large language model–powered multiagent system compared with physician preferences to improve the accuracy, relevance, and efficiency of order set optimization in clinical decision support.

## Introduction

Widespread implementation of electronic health records (EHRs) has led to the expansion of clinical decision support (CDS) systems.^1,2^ An important part of CDS is order sets within computerized clinician order entry systems, which are organized groups of orders or procedures compiled in 1 place, usually tailored to a specific condition, clinical process, or situation, such as a postoperative order set for knee arthroplasty.^3,4^ Well-designed order sets could improve efficiency in ordering, reduce errors, and improve adherence to clinical guidelines.^5,6,7,8^

Despite the benefits, order sets, similar to other CDS tools, also need systematic management and monitoring tools to detect malfunctions effectively.^9^ Currently, CDS experts often rely on third-party literature services for updates to clinical guidelines, introducing delays and missed updates. In addition, clinical evidence and practice shift over time.^10^ New drugs are introduced or new indications are found for existing drugs^11^; disease names and classifications are updated or revised^12,13^; and clinical procedures are added, changed, or reorganized.^14,15,16,17^ As an example, Vanderbilt University Medical Center (VUMC) currently maintains 1496 order sets for various clinical scenarios. Manual review of order sets to ensure they match up-to-date evidence is a resource-intensive process.

Large language models (LLMs) trained on large amounts of text have shown strong text-processing capabilities.^18^ In CDS, LLMs have been successfully used to critique alert criteria and summarize user comments on alerts.^19,20^ Multiagent systems consist of intelligent agents that collaborate to solve complex problems, and the capabilities of LLM agents are enhanced through iterative feedback and teamwork.^21,22^ They have demonstrated good performance in health monitoring and privacy-preserving clinical data sharing.^21,23,24^ This study aimed to develop and evaluate an LLM-powered multiagent system aligned with expert preferences to improve the accuracy, relevance, and efficiency of order set optimization in CDS.

## Methods

This cohort study was reported following the Strengthening the Reporting of Observational Studies in Epidemiology (STROBE) reporting guidelines.^25^ This study was performed at VUMC and approved as exempt from informed patient consent by the Vanderbilt Institutional Review Board because it involved secondary analysis of deidentified data and posed minimal risk to participants. The development of the multiagent system and the expert evaluations were performed in 2024. For the validation analysis, historical order data were extracted for the period between January 1 and December 31, 2024. We used Generative Pre-trained Transformer 4o (GPT-4o; OpenAI),^26^ where “o” denotes “omni,” a Microsoft Azure–hosted environment (Azure; Microsoft) approved for protected health information. Two experiments were conducted: (1) developing and evaluating a multiagent system to generate order set optimization suggestions and (2) developing and evaluating a customized filter to align these suggestions with expert preferences.

### Development and Evaluation of a Multiagent System

#### Multiagent System Development

**Figure 1.**
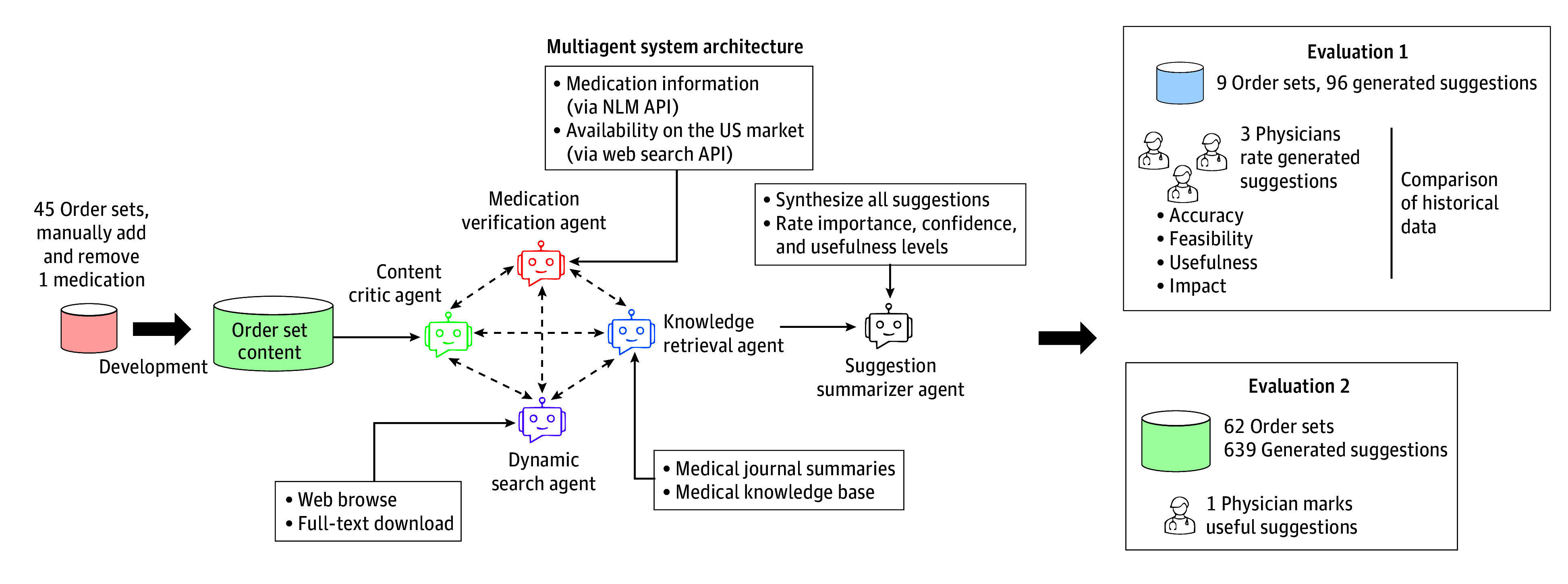
Overview of the Multiagent System Architecture and Evaluation Workflow The diagram illustrates the development and evaluation of the multiagent system. During development, 45 order sets were used. The system architecture consists of 5 agents—content critic, dynamic search, medication verification, knowledge retrieval, and suggestion summarizer—which interact to generate suggestions. The evaluation was conducted in 2 phases. Evaluation 1 involved 9 order sets, generating 96 suggestions rated by 3 physicians on accuracy, feasibility, usefulness, and impact. Evaluation 2 expanded to 62 order sets and 639 suggestions, with 1 physician identifying the useful ones. API indicates application program interface; NLM, National Library of Medicine.

We developed an LLM-powered multiagent system, including 5 agents (content critic agent, dynamic search agent, knowledge retrieval agent, medication verification agent, and the suggestion summarizer agent) (Figure 1). This architecture uses a retrieval-augmented generation process, where agents retrieve current external information to ground suggestions in clinical evidence. It could overcome knowledge cutoff limits of the base LLM, enabling adaptation to future medical changes without retraining. Our approach did not involve fine-tuning the base GPT-4o model. Instead, we used prompt engineering, where each agent was given a specific role and a detailed set of instructions. The agent interactions were designed as a structured sequential conversation facilitated by a manager agent. This manager selected a designated speaker at each step, whose output was then broadcast to all other agents to inform the next stage of the process. We implemented the multiagent conversation framework using an open-source tool (AutoGen; Microsoft).^27^

To optimize both the prompts and the agent interactions, we developed a refinement process using 45 VUMC order sets that were manually altered. For each set, we made controlled modifications—removing 1 correct medication and adding 1 unrelated medication—to create a reference standard with 2 expected suggestions. We then processed these sets through the multiagent system, reviewed the output, and iteratively adjusted the prompts and conversational flow to refine the system’s performance. The final prompts for each agent are available in eAppendix 1 in Supplement 1.

The content critic agent optimized order sets for clinical accuracy and relevance. It first reviewed the title to identify the disease and clinical scenario (eg, ambulatory, inpatient care) addressed by the order set. The agent then evaluated each included order to understand its structure and intended actions. For medication orders, it ensured that all relevant medications were explicitly listed by name, adding specific drugs if a general category (eg, pain medications) was used, and suggesting additional medications when necessary. Additionally, the agent identified outdated or inappropriate items. Through this detailed review process, the content critic agent enhanced the order set’s clinical accuracy using its own LLM. This initial analysis was subsequently combined with and validated against timely evidence provided by the knowledge retrieval agent.

The dynamic search agent played a key role in ensuring order sets were informed by the latest clinical knowledge. Using tools such as PubMed search and JavaScript, the agent retrieved full-text articles and clinical guidelines relevant to the order sets. These resources were saved in designated folders, enabling seamless follow-up retrieval and analysis by the knowledge retrieval agent.

The knowledge retrieval agent retrieved content from various sources, including *Journal Watch* from the *New England Journal of Medicine*, which summarizes newly published guidelines and articles from over 250 journals^28^; we extracted all summaries from January 2022 to July 2024. Other sources included the *Pocket Medicine* (7th edition), a reliable reference for accurate diagnosis and treatment planning in internal medicine, compiled by physicians at Massachusetts General Hospital.^29^ We also included StatPearls, a clinical support tool containing articles on diseases, drugs, and procedures, extracted from the National Center for Biotechnology Information Bookshelf.^30^ Another source was Vanderbilt Internal Medicine Residency Handbook (VIMBook), a yearly updated, peer-reviewed guide offering systems-based practice guidance at VUMC.^31^ The last source was PubMed articles and clinical guidelines previously saved by the dynamic search agent. This agent used the Chroma vector database to retrieve the 5 most relevant documents based on semantic similarity. This process enables the agent to generate targeted evidence-based suggestions, ensuring that order sets are based on comprehensive current clinical knowledge.

The medication verification agent used the RxNav application program interface (API) to extract medication class information and the Bing Search API to confirm current market availability. Finally, the suggestion summarizer agent synthesized the inputs from all agents to produce a final list of recommendations with confidence and importance scores.

#### Multiagent System Evaluation

In evaluation 1, 3 EHR software (Epic; Epic Systems)–certified physician builders (S.S.H., A.P.W., S.H.) rated suggestions from 9 order sets (selected based on 2023-2024 usage frequency) on a 1 to 5 Likert scale for accuracy, usefulness, feasibility, and impact, plus yes/no questions on redundancy and hallucination. The definition for each metric is provided in eTable 1 in Supplement 1. Informed consent (oral) was obtained from physician experts. No participants were lost to follow-up. We also analyzed historical data to understand how suggestions aligned with clinical practice. We extracted order data from the 2024 calendar year for encounters where each order set was used and calculated the 25th to 75th percentile usage counts for all associated orders. We then used this to assess alignment: a suggestion to add an item was considered aligned with frequent use if its count was above the 75th percentile, while a removal suggestion was considered aligned if its count was below the 25th percentile. Additionally, we performed a sensitivity analysis to determine the number of aligned suggestions across different percentile combinations (eg, 10th-90th, 35th-65th). In evaluation 2, 1 physician (S.S.H.) reviewed all suggestions for another set of 62 order sets to identify useful ones.

### Development and Evaluation of a Customized Filter for Aligning Generated Suggestions With Expert Preferences

This filter functions as a postprocessing layer to evaluate and rank suggestions after they have been generated. The filter was constructed and evaluated in the following steps.

**Figure 2.**
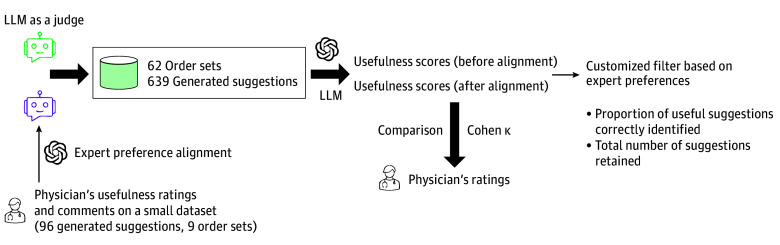
Workflow for Aligning AI-Generated Suggestions With Expert Physician Preferences This flowchart details the LLM-as-a-judge process for creating a customized filter. An LLM first assesses the usefulness of 639 suggestions from 62 order sets. These initial scores are then refined through an expert preference alignment process, which uses physician ratings and comments from a smaller dataset (96 suggestions). The agreement between the LLM's pre- and postalignment scores and the physician's ratings is measured using Cohen κ. This process results in a customized filter designed to retain a higher proportion of suggestions deemed useful by experts. AI indicates artificial intelligence; LLM, large language model.

The first step was the reference standard creation. A physician reviewed all 639 suggestions from evaluation 2 and assigned a binary rating (1 for useful, 0 for not useful). The second step was LLM-as-a-judge alignment and scoring. We used an LLM-as-a-judge approach (prompt in eAppendix 2 in Supplement 1) (Figure 2).^32^ A separate GPT-4o model was prompted to act as a judge and score the usefulness of each suggestion. To align this judge with expert preferences, we used few-shot prompting, in which annotated examples are provided in the prompt to guide the model’s responses, drawing from the 96 physician-annotated cases (evaluation 1). This postalignment judge then provided a new usefulness score for each suggestion. The third step was filter construction. We trained a logistic regression model using the postalignment usefulness score as the probability variable and the physician’s binary rating (from step 1) as the outcome. The fourth step was filter application and evaluation. The trained model was then applied as a filtering mechanism. By adjusting the model’s probability threshold, we could filter out suggestions with a low probability of being deemed useful by an expert. A comprehensive description of the model, threshold selection rationale, and uncertainty quantification is provided in eAppendix 4 in Supplement 1.

### Statistical Analysis

The Mann-Whitney *U* test was used to compare the pre- and postalignment usefulness scores against the physician’s evaluations.^33^ To measure the level of agreement between the LLM’s scores and the physician’s ratings, we calculated Cohen κ, a robust metric that accounts for agreement occurring by chance.^34^ Furthermore, logistic regression analysis was conducted to model the association between the postalignment usefulness scores and the physician’s binary usefulness rating from experiment 2 (outcome variable). Statistical analyses were conducted in Python version 3.11 (Python Software Foundation) using the packages scipy.stats, statsmodels, and scikit-learn, with a significance threshold of *P* < .001 (2-sided).

## Results

### Development and Evaluation of a Multiagent System

The knowledge base included 51 562 594 words (Table 1). From the 45 manually modified order sets, the system correctly generated suggestions for 41 (91%; 95% CI, 83%-99%) of the removed medications and 38 (84%; 95% CI, 74%-95%) of the added medications. This resulted in 88% accuracy on this specific development dataset, which we used to finalize the system’s prompt engineering before the main evaluation.

**Table 1.** Data Sources Used in the Knowledge Retrieval Agent

Source	Articles or pages, No.	Words, No.
NEJM Journal Watch (General Medicine) January 2022–July 2024	1275 articles	340 865
NEJM Journal Watch (Guideline Watch) January 2022–July 2024	90 articles	43 156
StatPearls^a^	9378 articles	50 919 241
*Pocket Medicine*	522 pages	133 903
Vanderbilt Internal Medicine Residency Handbook (VIMBook)	869 pages	125 429

Abbreviation: NEJM, *New England Journal of Medicine*.

^a^ Represents the entire corpus downloaded from the National Center for Biotechnology Information Bookshelf.

In evaluation 1 of experiment 1, across the 9 most frequently used order sets, the median number of generated suggestions per order set was 10 (IQR, 8-12). The medians for suggestions scoring 4 or higher were 5 (IQR, 5-6) for accuracy, 2 (IQR, 1-4) for usefulness, 1 (IQR, 0-3) for feasibility, and 1 (IQR, 0-2) for impact. eTable 2 in Supplement 1 details the number of suggestions scoring 4 or more for each metric.

**Figure 3.**
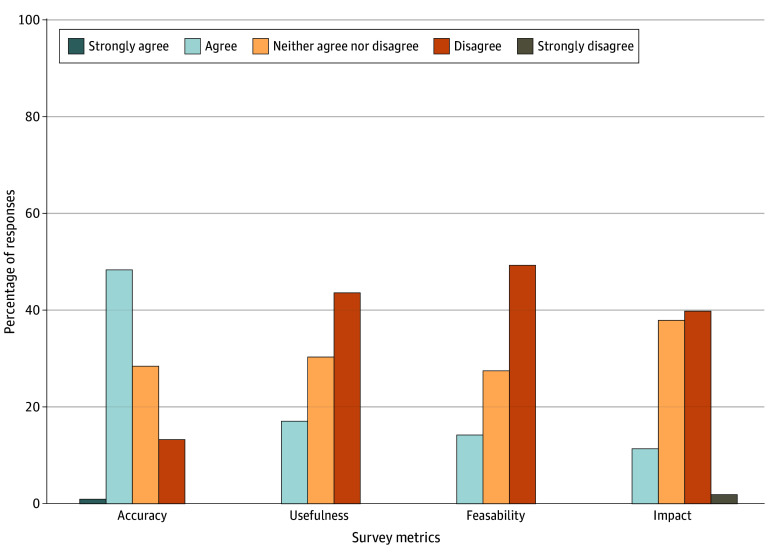
Physician Ratings of AI-Generated Suggestions Across 4 Key Metrics This divergent bar chart displays the distribution of ratings from physicians for 96 artificial intelligence (AI)–generated suggestions based on a 5-point scale from “strongly disagree” to “strongly agree.” The suggestions are evaluated across 4 metrics: impact, feasibility, usefulness, and accuracy. AI indicates artificial intelligence.

For the 96 suggestions generated for the initial 9 order sets, a high proportion were rated as accurate (54% scoring ≥4), while fewer were rated as highly useful (19%), feasible (16%), or direct impact (12%). Figure 3 illustrates the distribution of these ratings across metrics in a stacked bar chart. No suggestions were identified as hallucinations, although 11% were redundant. There were significant differences among rater scores (*P* < .001); for example, rater 1 and rater 3 most frequently agreed on a score of 2 (n = 229), whereas rater 1 assigned a score of 4 in 44 cases that rater 3 scored as 2. Confusion matrices for pairwise comparisons are provided in eFigure 1 in Supplement 1. Our analysis found that of 96 suggestions, 44 (46%; 95% CI, 36%-56%) aligned with historical ordering patterns (eg, suggestion to add apixaban to the order set aligned with data showing that apixaban was often ordered separately from the order set when the order set was used.) A sensitivity analysis showed the number of aligned suggestions changing from 18 (19%) at a 10th-90th threshold to 39 (41%) at a 30th-70th threshold (eTable 3 in Supplement 1). The estimated cost for analyzing a single order set ranges from $0.29 to $0.58, depending on the complexity of the order set and the number of agent interactions required.

In evaluation 2 of experiment 1, the multiagent system generated 639 suggestions across 62 order sets, with 52 sets receiving at least 1 useful suggestion. The median of the number of useful suggestions at the order set level was 2 (IQR, 1-3). Specifically, 4 order sets had up to 5 useful suggestions and 8 order sets had 4. Overall, 122 suggestions (19%; 95% CI, 16%-22%) were rated as useful. Table 2 lists examples of useful suggestions. For example, we submitted a ticket to modify the total knee arthroplasty postoperative focused orders order set based on the suggestion “Add Apixaban (Eliquis) as an alternative anticoagulant option.” A detailed breakdown of these suggestions by clinical scenario and suggestion type is provided in eTable 4 (clinical scenario) and eTable 5 (suggestion type) in Supplement 1.

**Table 2.** Examples of Useful Suggestions

Order set	Generated suggestion		Generated rationale
	Text	Usefulness	
Total knee arthroplasty postoperative focused orders	Add apixaban (Eliquis) as an alternative anticoagulant option.	4/5^a^	Apixaban is a well-tolerated anticoagulant that can be used as an alternative to other anticoagulants in postoperative care.
Adult hemodialysis orders	Evaluate the necessity of sodium bicarbonate 8.4 injection for blood pressure support, as it is typically used for metabolic acidosis.	3/5^a^	Sodium bicarbonate is not typically used for blood pressure support, and its inclusion should be reviewed for appropriateness.
Adult post–lung transplant routine clinic protocol	Add glycated hemoglobin for patients with diabetes or at risk of developing diabetes due to immunosuppressive therapy.	4/5^a^	Monitoring long-term glucose control is important in managing diabetes.
Neurology admission orders	Consider adding a lipid panel for patients at risk for stroke or cardiovascular disease.	4/5^a^	A lipid panel can help assess cardiovascular risk, which is relevant for neurology patients.
Neurology admission orders	Consider adding carotid ultrasound for patients at risk for stroke or transient ischemic attacks.	4/5^a^	Carotid ultrasound can help assess stroke risk and guide management decisions.
Low back pain care path—chronic	Add acetaminophen to the medication list.	1^b^	Acetaminophen is often used as a first-line treatment for pain management and can be used in conjunction with NSAIDs.

Abbreviation: NSAID, nonsteroidal anti-inflammatory drug.

^a^ Usefulness rated on a 1-5 Likert scale (from evaluation 1).

^b^ Usefulness rated on a binary scale where 1 indicates “useful” (from evaluation 2).

### Development and Evaluation of a Customized Filter for Aligning Generated Suggestions With Expert Preferences

Before alignment, the Mann-Whitney *U* test showed no association between LLM-generated usefulness scores and rater ratings. After alignment, an association was found between updated scores and ratings. Cohen κ improved from 0.06 (poor agreement) to 0.41 (moderate agreement) after alignment (eFigure 2 in Supplement 1). The finding for updated usefulness scores and rater ratings was significant using the logistic regression model: χ^2^_1_ = 71.36. The updated usefulness score was positively associated with the likelihood of the outcome (β = 0.05; 95% CI, 0.03-0.06). Using this model as a filter, a threshold of 0.11 retained 453 suggestions (a 29% reduction) while preserving 92% of useful suggestions. Detailed results are shown in eTable 6 in Supplement 1.

## Discussion

The results of this cohort study suggest the feasibility of an LLM-powered multiagent system for optimizing order sets. The primary value of this system is its ability to provide a systematic and scalable evidence-grounded foundation for a task that is traditionally a resource-intensive manual review. By automatically generating a targeted list of suggestions, the system shifts the expert’s role from manual discovery to efficient validation, addressing the challenges of scale and currency inherent in the manual process.

This system allowed for customization by integrating external resources based on specific needs, and tailoring prompts or system architecture to achieve diverse optimization goals. Such flexibility enabled CDS experts at different institutions to adapt the multiagent system based on their local conditions. The current manual process of optimizing order sets is highly resource intensive. While experts might be able to identify incorrect items in a long order set, determining what was missing was far more challenging, particularly given the continual updates in clinical evidence and the complexity of the order set in both content and in structure. A multiagent system, however, systematically reviewed every item in every order set and compared it with external evidence, regardless of the number of order sets.

This study demonstrated the ability of an LLM-powered multiagent system to generate suggestions that are relevant to patient care. For example, in the total knee arthroplasty postoperative focused orders order set, the system suggested adding apixaban as an alternative anticoagulant option. This was rated as highly useful because it is clinically appropriate, aligns with current evidence, and represents a direct actionable improvement to the order set. As a contrasting example, the suggestion to add a baseline laboratory panel to the behavioral health admission orders received a low usefulness rating. Reviewer feedback pointed out the suggestion’s ambiguity; it was unclear whether this particular order matched the correct clinical workflow for users of the order set. Additionally, some suggestions, while less directly useful, could inspire experts and might also be highly relevant to patient care. For instance, in the adult post–lung transplant routine clinic protocol order set, the system suggested considering medications such as metformin or insulin for managing posttransplant diabetes. One physician noted that, although prescribing metformin might not be directly applicable, the suggestion raised a question about whether glycated hemoglobin testing should be included in the order set.

The modest useful suggestion rate (19%) highlights a key challenge: many suggestions, while factually correct, lacked the specific clinical context to be deemed useful. This result is partly due to the system’s design, which prioritized generating a larger pool of suggestions to avoid omitting valuable insights. This may be due to factors such as order set complexity and maturity, since frequently used sets often have been completed multiple rounds of manual optimization, leaving limited room for further improvement. Future research could therefore focus on integrating a deeper understanding of clinical workflows and institutional priorities to better align technical accuracy with practical utility. Importantly, the retrieval-augmented generation architecture likely was a factor associated with the absence of factual hallucinations.

Relying solely on an LLM to evaluate suggestion usefulness is insufficient. Optimizing order sets is a complex task, requiring a deep understanding of clinical workflows and domain-specific knowledge. Physicians noted difficulties rating suggestions for highly specific scenarios, leading to discrepancies between LLM scores and their ratings. This observation is matched with the initial inconsistency between LLM-generated usefulness scores and physician ratings. Our findings showed an association between incorporating a small set of physician ratings and the accuracy of LLM-generated usefulness scores. After alignment, Cohen κ improved from poor to moderate agreement across a large dataset. It is consistent with previous findings showing enhanced GPT-4o performance in a triage task after expert alignment.^35^

A proposed clinical implementation model, illustrated by our prototypes in eFigures 3 and 4 in Supplement 1, was designed to address practical challenges. The proposed workflow integration involves periodic automated suggestion generation, which a CDS expert triages and assigns to relevant physician specialists for an efficient asynchronous review. A step-by-step description is available in eAppendix 3 in Supplement 1.

### Limitations

This study has several limitations. Conducted at a single academic center, the generalizability of the findings may be limited, although the use of the EHR infrastructure enhances technical transferability. The high-usage order sets selected may not be representative, and the small number of physician builder evaluators may not reflect all clinician perspectives. Our design did not systematically quantify false negatives, nor did we directly evaluate the traceability of suggestions to their source. Future research could compare this multiagent architecture with a single-agent system, enhance the reasoning capabilities and knowledge base,^36^ and implement the framework detailed in eAppendix 5 in Supplement 1 to quantitatively measure suggestion faithfulness, building clinician trust and facilitating EHR integration.

## Conclusions

In this cohort study, leveraging LLMs and multiagent systems provided a systematic and scalable approach to optimizing order sets but faced challenges in addressing specific clinical needs. Alignment with a small set of expert ratings was associated with stronger LLM evaluation capabilities. Future research could focus on refining reasoning capabilities, expanding the knowledge base, and facilitating the integration of useful suggestions into EHRs, while actively engaging end-users of order sets as human reviewers supported by artificial intelligence.

## Notes

Supplement 1. **eTable 1.** Definitions for suggestion rating criteria **eFigure 1.** Confusion matrices for pairwise comparisons **eAppendix 1.** Prompts for each agent in the multi-agent system **eAppendix 2.** Prompts for evaluating the usefulness of suggestions **eTable 2.** Number of suggestions scoring 4 or higher for each metric at the order set level **eFigure 2.** Comparison of Cohen’s Kappa values across various thresholds before and after alignment **eTable 3.** Sensitivity analysis of percentile thresholds for suggestion validation **eTable 4.** Distribution and usefulness of generated suggestions by clinical scenario **eTable 5.** Distribution and usefulness of generated suggestions by suggestion type **eTable 6.** Performance of the filter on the multi-agent system across different probability thresholds **eFigure 3.** Physician-facing user interface prototype for reviewing generated suggestions. The interface allows physicians to evaluate suggestions for order sets and provide feedback **eFigure 4.** CDS expert-facing user interface prototype for overseeing the review process **eAppendix 3**. Proposed real-world implementation and maintenance workflow **eAppendix 4**. Detailed methodology and validation of the logistic regression filter **eAppendix 5.** Proposed framework for quantitatively evaluating the evidence traceability of generated suggestions

Supplement 2.
Data Sharing Statement
