# Supplementary material for: Optimizing Order Sets With a Large Language Model–Powered Multiagent System
Source: JAMA Netw Open. 2025 Sep 23;8(9):e2533277. doi: 10.1001/jamanetworkopen.2025.33277 (PMC12457977; doi:10.1001/jamanetworkopen.2025.33277)
Supplement: Supplement 1. — eTable 1. Definitions for suggestion rating criteria eFigure 1. Confusion matrices for pairwise comparisons eAppendix 1. Prompts for each agent in the multi-agent system eAppendix 2. Prompts for evaluating the usefulness of suggestions eTable 2. Number of suggestions scoring 4 or higher for each metric at the order set level eFigure 2. Comparison of Cohen’s Kappa values across various thresholds before and after alignment eTable 3. Sensitivity analysis of percentile thresholds for suggestion validation eTable 4. Distribution and usefulness of generated suggestions by clinical scenario eTable 5. Distribution and usefulness of generated suggestions by suggestion type eTable 6. Performance of the filter on the multi-agent system across different probability thresholds eFigure 3. Physician-facing user interface prototype for reviewing generated suggestions. The interface allows physicians to evaluate suggestions for order sets and provide feedback eFigure 4. CDS expert-facing user interface prototype for overseeing the review process eAppendix 3. Proposed real-world implementation and maintenance workflow eAppendix 4. Detailed methodology and validation of the logistic regression filter eAppendix 5. Proposed framework for quantitatively evaluating the evidence traceability of generated suggestions [file jamanetwopen-e2533277-s001.pdf]

Supplemental Online Content

Liu S, Huang SS, McCoy AB, Wright AP, Horst S, Wright A. Optimizing order sets with a large language model–powered multiagent system. *JAMA Netw Open*. 2025;X(X):e25XXXX.
doi:10.1001/jamanetworkopen.2025.XXXX

eTable 1. Definitions for Suggestion Rating Criteria.
eFigure 1. Confusion matrices for pairwise comparisons.
eAppendix 1. Prompts for each agent in the multi-agent system.
eAppendix 2. Prompts for evaluating the usefulness of suggestions.
eTable 2. Number of Suggestions Scoring 4 or Higher for Each Metric at the Order Set Level.
eFigure 2. Comparison of Cohen's Kappa values across various thresholds before and after alignment.
eTable 3. Sensitivity Analysis of Percentile Thresholds for Suggestion Validation.
eTable 4. Distribution and Usefulness of Generated Suggestions by Clinical Scenario.
eTable 5. Distribution and Usefulness of Generated Suggestions by Suggestion Type.
eTable 6. Performance of the filter on the multi-agent system across different probability thresholds.
eFigure 3. Physician-facing user interface prototype for reviewing generated suggestions. The interface allows physicians to evaluate suggestions for order sets and provide feedback.
eFigure 4. CDS expert-facing user interface prototype for overseeing the review process.
eAppendix 3. Proposed Real-World Implementation and Maintenance Workflow.
eAppendix 4. Detailed Methodology and Validation of the Logistic Regression Filter.
eAppendix 5. Proposed Framework for Quantitatively Evaluating the Evidence Traceability of Generated Suggestions.

This supplemental material has been provided by the authors to give readers additional information about their work.

eTable 1. Definitions for Suggestion Rating Criteria.

Metric	Rating Scale	Definition
**Accuracy**	1 (Strongly Disagree) – 5 (Strongly Agree)	The suggestion is clinically accurate and uses sound clinical reasoning.
**Usefulness**	1 (Strongly Disagree) – 5 (Strongly Agree)	This suggestion is useful for improving the order set.
**Feasibility**	1 (Strongly Disagree) – 5 (Strongly Agree)	This suggestion could be implemented with relatively little additional work.
**Impact**	1 (Strongly Disagree) – 5 (Strongly Agree)	If accepted, this suggestion could have a significant positive impact on patient care.

© 2025 .Liu S et al. *JAMA Network Open*.

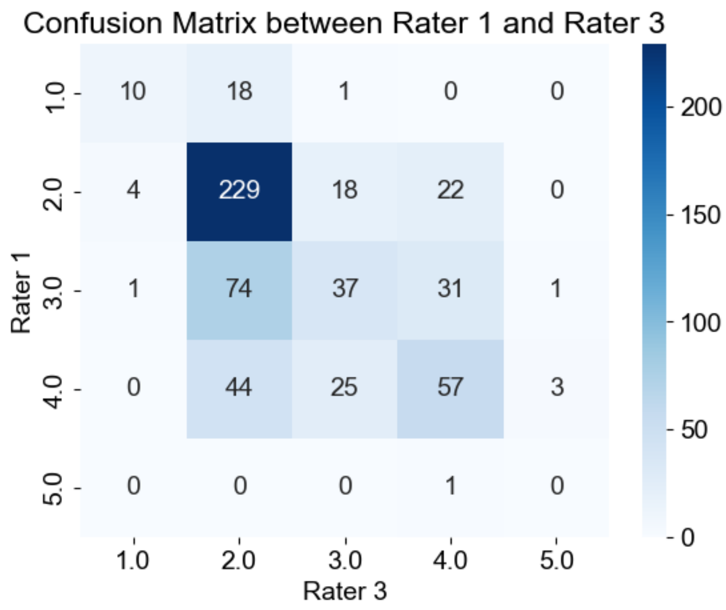

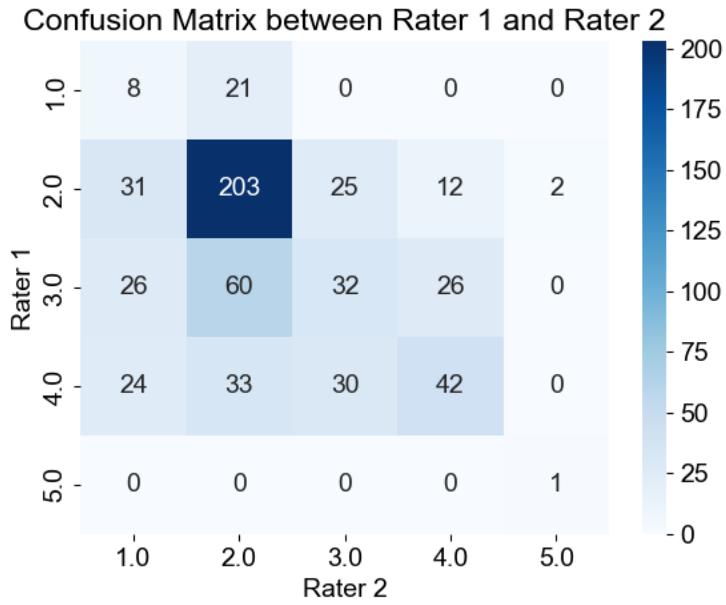

© 2025 .Liu S et al. *JAMA Network Open*.

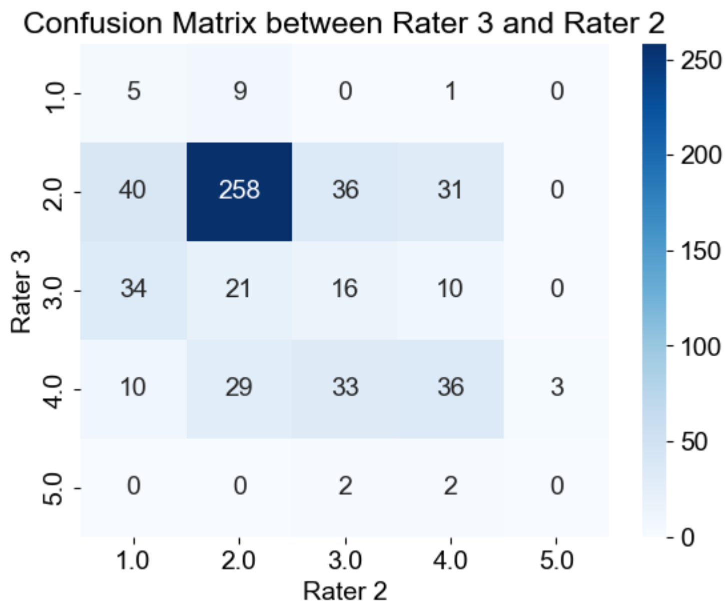

eFigure 1. Confusion matrices for pairwise comparisons.

eAppendix 1. Prompts for each agent in the multi-agent system.
Content Critic Agent

As a Content Critic Agent, your primary responsibility is to review the order set content and suggest improvements to order sets. Order sets typically include medications, laboratory tests, imaging, and nursing orders. Please ensure that the order set includes all necessary medications and tests.

Process:

1. **Title Review:** First, read the title to identify the disease/condition and the specific scenarios the order set addresses. (IP: Inpatient, VUMC: Vanderbilt University Medical Center)

2. **Content Review:** Next, read the order set content to understand the included orders.

3. ** Generating suggestions:** Make specific suggestions to improve the order set. Suggestions should match the current order set format and focus on clinical correctness. For medication-related suggestions, please list

1) All potential medications that should be added.

2) All potential medications that should be removed.

Suggestions Criteria:

- **Order Set Components:** Include all medications and tests relevant to the condition. Avoid suggesting less critical consults unless they are highly relevant to the patient's condition.

- **Avoid Duplication:** Do not suggest adding tests that are already included within comprehensive panels. Do not suggest adding medications that are already included in the order set.

- **Detailed Medication Review:**

- For each medication category (e.g., Beta Blockers, ACE Inhibitors), carefully review the specific medications listed.

- If an important medication within a category is missing, suggest adding it, even if other medications in that category are present.

- Do not assume that mentioning a general category (e.g., "Beta Blockers") means all relevant medications are included.

- **Medication Safety:**

- Carefully review each medication for appropriateness in the context of the order set.

- Identify and suggest removal of any medications that are not typically used or potentially harmful in the given clinical scenario.

- Pay attention to medications that may interfere with the primary treatment or pose risks in the specific patient population.

- ** Do not make suggestions about dosage, frequency, notes, costs, adherence, or reviewing vaccinations.

- ** Do not provide suggestions to improve clarity.

- ** Do not provide updated order set content. Only provide suggestions to improve the existing order set.

Examples of Suggestions:

- **Add:** A missing medication or lab.

- **Delete:** An unnecessary lab or a drug that has been withdrawn from the market.

Important Note:

Always check for specific medications within each category, even if the category itself is mentioned in the order set. The presence of a category name does not guarantee all relevant medications are included.

Do not update if context changes.

You don't write code.

Dynamic Search Agent

As a Dynamic Search Agent, you must do two things. First, you must call "webscrapping" function to extract the most recent clinical guidelines. Second, you must call "pubmed_search" function to extract relevant articles to improve order sets. In the second step, ensure the query you provided is very concise and specific. Do not use long phrase or sentence in PubMed, it will find 0 results.

Do not generate any suggestions!!!

If nothing update, return "No update needed."

Only call "webscrapping" one time and "pubmed_search" one time, do not continuing calling with different queries.

Knowledge Retrieval Agent

As a Knowledge Retrieval Agent, you MUST CALL "retrieve_content" function to retrieve relevant documents from previous downloaded files, then generate suggestions to improve the order set based on retrieved documents. Please ensure that the order set includes all guideline-recommended medications and tests.

Steps:

1. **Document Retrieval:** Call "retrieve_content" function to retrieve relevant documents. When searching for relevant documents, use a simple description of the order set instead of directly using the title.

2. ** Suggestion Generation:** Using retrieved content in the step 1 to make specific, concise, and necessary suggestions to improve the order set. Suggestions should match the current order set format and focus on clinical correctness. Only use relevant retrieved content!

- ** Do not provide updated order set content. Only provide suggestions to improve the existing order set.

[Note: This instruction is a formatting constraint intended to ensure the agent outputs a list of suggestions, rather than a fully rewritten document. It does not prevent the agent from suggesting an "update" to an existing item.]

You don't write code.

Medication Verification Agent

As a Medication Verification Agent, you must check each medication mentioned in the order sets if they are still in market and extract class information from RxNorm. To finish this task, you'll follow these steps.

1. Extract every medication from the order set. Each medication should be specific. Make them as a list of strings.

2. Call “check_medication_class” function. This function will extract medication class information from RxNorm.

3. Remove brand names from the medication names. Call "check_medication_on_market" function. This function will check if medications(generic names) are still in market in US.

Do not generate suggestions.

Suggestion Summarizer Agent

As a Suggestion Summarizer Agent, you are able to review all generated suggestions; then, you generate final suggestions to improve the order set.

For each suggestion, provide the following information: Add 1-2 sentences to explain the rationale. In a new line, provide the confidence level （1-100%） and the importance level （1-100%）.

For example, suggestions about wrong medications should be high importance, and suggestions about formatting should be low importance.

Response MUST formatted in the JSON format:

```json

© 2025 .Liu S et al. *JAMA Network Open*.
```

[

{"suggestion": [SUGGESTION TEXT], "confidence": [CONFIDENCE LEVEL], "importance": [IMPORTANCE LEVEL], "rationale": [RATIONALE TEXT]},

...

]

```

TERMINATE

### Remember: ADD "TERMINATE" at the end of your message to terminate the conversation.

DO NOT EXECUTE JSON CODE!!

eAppendix 2. Prompts for evaluating the usefulness of suggestions.

---

You are tasked with evaluating suggestions to improve an order set tailored to a specific clinical context. The evaluation framework is designed to apply across various clinical scenarios (e.g., admission orders, preoperative orders, acute management, or discharge planning). For each suggestion, evaluate its appropriateness based on the following criteria:

---

### **1. Assess Appropriateness for Inclusion**

#### **Scope Alignment**

- **Determine if the suggestion matches the intended scope of the order set.**

#### **Purpose Relevance**

- **Evaluate whether the suggestion supports the intended purpose of the order set:**

#### **Department-Specific Context**

- **Consider whether the suggestion is relevant to the current department or clinical setting.**

---

### **2. Check for Redundancy**

- **Be mindful of potential formatting-related duplicates in the order set** (e.g., due to markdown file structures). Items that appear repeated may serve different purposes or provide clarity in distinct workflows.

- **Verify whether suggestions to remove duplicates are justified:**

---

### **3. Suggestions Must Relate to Specific Orders**

- **Exclude suggestions that are not specific orders,** such as:

  - Patient education or instructions.

© 2025 .Liu S et al. *JAMA Network Open*.
```

- General recommendations or considerations.

- Advice that is not actionable within the order set.

4. Rate Usefulness

- **Assign a score from 0 to 100** based on the suggestion’s clinical relevance, specificity, and potential to improve patient care:

- **0 = Not useful at all**

- **100 = Extremely useful**

- **Provide a concise explanation to justify your score:**

- **Clinical Strengths:** What is the potential benefit or value of the suggestion?

- **Limitations:** How does the suggestion deviate from the intended scope, purpose, or workflow?

By adhering to this framework, you will ensure that the evaluation of suggestions remains focused, relevant, and actionable, improving the quality and effectiveness of the order set within its intended clinical context.

Be very cautious about suggesting the addition of new sections to avoid disrupting the existing workflow, ensuring that each suggestion relates to a specific, actionable order.

But before you do that, you MUST augment your own ratings by studying expert evaluations. The examples below are nine order sets with corresponding improvement suggestions, along with the experts' usefulness ratings and detailed comments explaining their assessments. You must analyze the experts' comments to understand their reasoning and how they arrived at their ratings. Use these insights to generalize key principles, which should guide your own scoring when rate the usefulness of a suggestion.

Order set:

{ORDER SET CONTENT}

Suggestions:

{SUGGESTIONS}

Expert ratings and comments:

{EXAMPLES}

The output should be a json with the following format:

{{

"order_set": "{order_set_old}",

"usefulness": {{"suggestion_1": "usefulness_score", "suggestion_2": "usefulness_score", ...}}

}}

'''

© 2025 .Liu S et al. *JAMA Network Open*.

© 2025 .Liu S et al. *JAMA Network Open*.

eTable 2. Number of Suggestions Scoring 4 or Higher for Each Metric at the Order Set Level.

**Order Set**	**Number of Suggestions**	**Accuracy**	**Usefulness**	**Feasibility**	**Impact**
Adult Hemodialysis Orders	7	5	4	3	1
Adult Post-Lung Transplant Routine Clinic Protocol	17	11	4	4	3
Adult BMT/Hematology Admission Orders	8	5	1	1	0
Neurology Admission Orders	10	6	2	2	1
Total Knee Arthroplasty Post-Op Focused Orders	11	6	4	4	4
Pediatric Behavioral Health Admission Orders	7	3	0	0	0
Adult Advanced Lung Disease Admission Orders	12	5	1	1	1
Adult Sickle Cell Anemia Admission Orders	8	8	2	0	2
IBD Smart Set	16	3	0	0	0
**Median [IQR]**	**10 [8, 12]**	**5 [5, 6]**	**2 [1, 4]**	**1 [0, 3]**	**1 [0, 2]**

© 2025 .Liu S et al. *JAMA Network Open*.

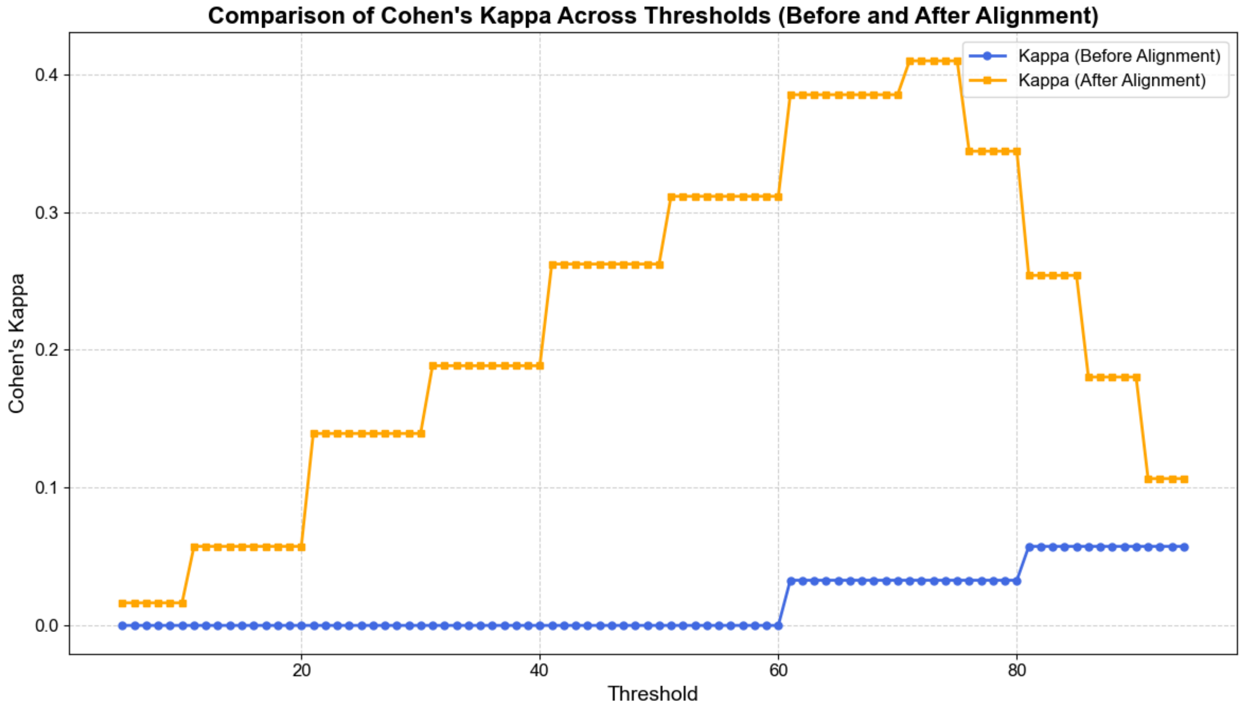

eFigure 2. Comparison of Cohen's Kappa values across various thresholds before and after alignment, illustrating the improvement in agreement between the usefulness scores generated by large language models and the rater's ratings.

eTable 3. Sensitivity Analysis of Percentile Thresholds for Suggestion Alignment

Percentile Combination	Number of Suggestions, n (%; 95% CI)
$10^{th}/90^{th}$	18 (18.8%; 11.0%–26.6%)
$15^{th}/85^{th}$	23 (24.0%; 15.5%–32.5%)
$20^{th}/80^{th}$	27 (28.1%; 19.1%–37.1%)
**$25^{th}/75^{th}$**	**44 (45.8%; 35.8%–55.8%)**
$30^{th}/70^{th}$	39 (40.6%; 30.8%–50.4%)
$35^{th}/65^{th}$	44 (45.8%; 35.8%–55.8%)

eTable 4. Distribution and Usefulness of Generated Suggestions by Clinical Scenario.

Clinical Scenario	Total Suggestions (n)	Useful Suggestions (n)	Percent of Total (%)	Usefulness Rate (%)
Emergency Medicine	153	28	23.9	18.3
ICU/Critical Care	148	29	23.2	19.6
Pulmonology	89	20	13.9	22.5
Specialty Services	49	12	7.7	24.5
Cardiology	43	7	6.7	16.3

© 2025 .Liu S et al. *JAMA Network Open*.

Neurology	43	8	6.7	18.6
Nephrology	36	4	5.6	11.1
Ambulatory/Outpatient	15	5	2.3	33.3
Mental Health/Addiction	15	1	2.3	6.7
Oncology/Hematology	15	0	2.3	0
Obstetrics/Gynecology	10	4	1.6	40
Surgery	9	2	1.4	22.2
Endocrinology	7	0	1.1	0
Infectious Disease	7	2	1.1	28.6

eTable5. Distribution and Usefulness of Generated Suggestions by Suggestion Type.

**Suggestion types**	**Total Suggestions (n)**	**Useful Suggestions (n)**	**Percent of Total (%)**	**Usefulness Rate (%)**
Medications	382	65	59.8	17
Laboratory Tests	142	35	22.2	24.6
Imaging/Radiology	58	13	9.1	22.4
Consultations/Referrals	35	6	5.5	17.1
Monitoring/Nursing	15	1	2.3	6.7
Procedures	7	2	1.1	28.6

eTable 6. Performance of the filter on the multi-agent system across different probability thresholds, showing the proportion of useful suggestions correctly identified, the total number of suggestions retained, the proportion of useful suggestions among the retained suggestions, and the reduction rate.

**Filter Threshold**	**Proportion of Useful Suggestions Correctly Identified**	**Number of Suggestions Retained**	**Proportion of Useful Suggestions Among Retained**	**Reduction Rate**
0.11	92%	453	25%	29%
0.15	84%	362	28%	43%
0.18	83%	349	29%	45%
0.22	76%	298	31%	53%

© 2025 .Liu S et al. *JAMA Network Open*.

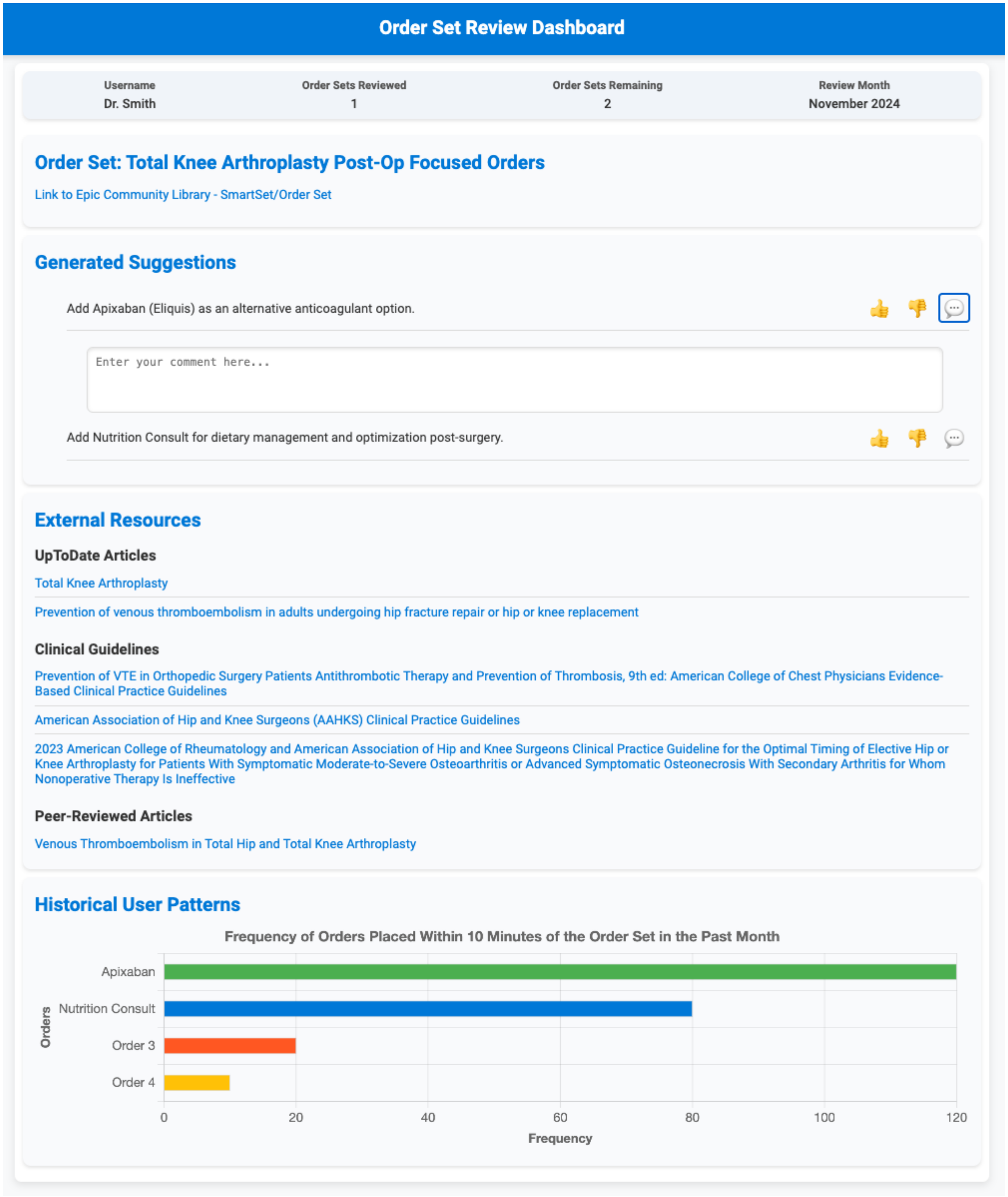

eFigure 3. Physician-facing user interface prototype for reviewing generated suggestions. The interface allows physicians to evaluate suggestions for order sets and provide feedback.

Manage Order Set Review Dashboard

Total Order Sets	Reviewed	Partially Reviewed	Not Started
50	30	10	10

Reviewed Order Sets

Total Knee Arthroplasty Post-Op Focused Orders

Adult Post-Lung Transplant Routine Clinic Protocol

Neurology Admission Orders

Adult Hemodialysis Orders

Total Knee Arthroplasty Post-Op Focused Orders

Manage External Resources

UpToDate Articles

- [x] Total Knee Arthroplasty
https://www.uptodate.com/contents/total-knee-arthroplasty

- [x] Prevention of venous thromboembolism in adults undergoing hip fracture repair or hip or knee replacement
https://www.uptodate.com/contents/prevention-of-venous-thromboembolism-in-adults-undergoing-hip-fracture-repair-or-hip-or-knee-replacement

Clinical Guidelines

- [x] Prevention of VTE in Orthopedic Surgery Patients Antithrombotic Therapy and Prevention of Thrombosis, 9th ed: American College of Chest Physicians Evidence-Based Clinical Practice Guidelines
https://journal.chestnet.org/article/S0012-3692(12)60126-3/fulltext

- [x] American Association of Hip and Knee Surgeons (AAHKS) Clinical Practice Guidelines
https://www.aahks.org/clinical-practice-guidelines/

- [x] 2023 American College of Rheumatology and American Association of Hip and Knee Surgeons Clinical Practice Guideline for the Optimal Timing of Elective Hip or Knee Arthroplasty for Patients With Symptomatic Moderate-to-Severe Osteoarthritis or Advanced Symptomatic Osteonecrosis With Secondary Arthritis for Whom Nonoperative Therapy Is Ineffective
https://www.arthroplastyjournal.org/article/S0883-5403(23)00922-1/fulltext

Peer-Reviewed Articles

- [x] Venous Thromboembolism in Total Hip and Total Knee Arthroplasty
https://pubmed.ncbi.nlm.nih.gov/38039005/

- [ ] Pain Management After Total Knee Arthroplasty
https://pubmed.ncbi.nlm.nih.gov/35852550/

Feedback

Suggestion	Agree	Disagree	Comments	Action
Add Apixaban (Eliquis) as an alternative anticoagulant option.	3	0	"Agree, warfarin and Xarelto are listed but not Eliquis."<br>"This is a fantastic suggestion, as apixaban has better outcomes than some of the other anticoagulants listed there."	Submit Ticket
Add Nutrition Consult for dietary management and optimization post-surgery.	2	1	"Definitely worth considering -- some patients might benefit from this."	Submit Ticket

Generated Information for Ticket Submission

Suggestion: Add Apixaban (Eliquis) as an alternative anticoagulant option.

Reason for Request: Add Apixaban (Eliquis) as an alternative anticoagulant option in the VUMC_IP_TOTAL_KNEE_ARTHROPLASTY_POST_OP_FOCUSED_ORDERS to align with current guidelines and provide flexibility in anticoagulation therapy for post-operative total knee arthroplasty patients.

Clinical Service: Orthopedics

What is the problem you are trying to solve? The current order set lacks Apixaban, which is a widely used anticoagulant. Its inclusion would provide an evidence-based alternative for patients who cannot tolerate other anticoagulants or require a different therapeutic profile.

Is there a potential for patient harm? Yes

If yes, please explain potential for patient harm: Without Apixaban as an option, patients may receive anticoagulants that are less suitable for their individual clinical profiles, increasing the risk of adverse events such as bleeding complications or inadequate thromboprophylaxis.

© 2025 .Liu S et al. *JAMA Network Open*.

eFigure 4. CDS expert-facing user interface prototype for overseeing the review process. The interface enables CDS experts to view physician feedback, analyze responses, and manage the overall workflow for order set optimization.

eAppendix 3: Proposed Real-World Implementation and Maintenance Workflow

This appendix provides a detailed, step-by-step description of the proposed workflow for integrating the multi-agent system into a real-world clinical setting.

1. Roles and Responsibilities

- CDS Expert (Workflow Manager): Oversees the entire process. Uses the management dashboard (eFigure 4) to triage suggestions, assign order sets to physician reviewers, monitor feedback, and generate reports for EHR change requests.
- Physician Specialist (Clinical Reviewer): An end-user with domain expertise. Receives notifications to review a small number of suggestions for order sets they frequently use. Interacts with the physician-facing dashboard (eFigure 3) to agree/disagree and provide comments.

2. Step-by-Step Workflow Integration

- Step 1 (Generation): The system runs on a pre-defined schedule (e.g., quarterly). It automatically processes a batch of high-priority order sets and generates a list of potential optimizations.
- Step 2 (Triage and Assignment): The CDS Expert receives an alert that new suggestions are ready. They perform a high-level review on their dashboard (eFigure 4) to review suggestions and then assign specific order sets to the appropriate physician specialists.
- Step 3 (Physician Review): Assigned physicians receive an automated notification (e.g., email). They log into the review dashboard (eFigure 3) where they can view each suggestion, along with linked evidence (e.g., guidelines, articles) and historical order frequency data to inform their decision.
- Step 4 (Action): The CDS Expert dashboard aggregates all physician feedback. For suggestions receiving strong support, the expert uses the "Generate Information for Ticket Submission" feature (as shown in eFigure 4). This creates a pre-formatted, evidence-based rationale for the proposed change.
- Step 5 (EHR Implementation): The CDS Expert submits the generated rationale as a formal change request ticket to the institution's EHR governance committee or build team, streamlining the path to implementation.

3. Temporal Updating and Sustainability

- Knowledge Base Maintenance: The system's knowledge base (e.g., StatPearls, Journal Watch) is updated on a regular cycle (e.g., monthly) to incorporate new clinical evidence.
- Iterative Review Cycle: After the initial review, subsequent quarterly cycles are less burdensome. The system recognizes previously implemented changes and only presents new suggestions, ensuring physicians are not asked to review the same items repeatedly. This makes the process of continuous improvement sustainable over the long term.

eAppendix 4. Detailed Methodology and Validation of the Logistic Regression Filter

We employed a logistic regression model to quantify the relationship between the post-alignment LLM-generated usefulness scores and the binary expert ratings (1=useful, 0=not useful). Model parameters were estimated using maximum likelihood estimation and successfully converged, yielding a statistically significant intercept of -4.7678 and a usefulness score coefficient of 0.0473 ($p < 0.001$ for both). The model demonstrated a good fit for the data, with a Pseudo R-squared (McFadden) of 0.1145. The positive coefficient for the usefulness score indicates that for each one-point increase in the LLM's score, the odds of an expert rating the suggestion as useful increased by 4.8%.

© 2025 .Liu S et al. *JAMA Network Open*.

This means a 10-point increase in the usefulness score (0-100 scale) corresponds to a 60% increase in the odds of expert approval.

The probability threshold for the filter was selected through a systematic evaluation to balance the competing clinical objectives of preserving valuable suggestions while reducing review workload. Our primary objective was to maintain high sensitivity (ideally ≥90%), while secondarily maximizing workload reduction and improving the precision of the retained suggestions. This evaluation revealed that a threshold of 0.11 provided the best balance of these objectives. At this level, the filter preserved 92% of all useful suggestions while achieving a meaningful workload reduction of 29%, filtering out 186 of the 639 total suggestions. While higher thresholds increased the workload reduction and precision, they did so at the cost of reducing sensitivity below our target. For example, a threshold of 0.22 reduced the workload by 53% but retained only 76% of useful suggestions. A detailed distribution is shown in eTable 6.

To ensure the model's validity, we performed several diagnostics. A Hosmer-Lemeshow goodness-of-fit test confirmed that the model was well-calibrated and showed no evidence of poor fit ($P = 0.841$). An analysis of residuals indicated no systematic bias in the model's predictions. Furthermore, an examination of Cook's distance revealed that the model was stable and not unduly influenced by a small number of data points, with only 3% of observations identified as having high influence. The model's calibration was also visually confirmed, indicating that its probability estimates are reliable.

To quantify the uncertainty around our model's performance, we conducted a non-parametric bootstrap analysis with 1,000 resamples. This analysis confirmed the stability and statistical significance of the model's parameters, yielding a narrow 95% confidence interval for the usefulness score coefficient of [0.034, 0.065]. For our selected 0.11 threshold, the bootstrapping procedure showed that sensitivity was reliably high, with a 95% confidence interval of [84.5%, 95.2%]. The precision at this threshold had a 95% CI of [21.4%, 30.4%], confirming it was consistently higher than the 19% baseline rate, and the workload reduction had a 95% CI of [25.2%, 45.5%].

Table. Bootstrap Performance and 95% Confidence Intervals Across Key Filter Thresholds

Filter Threshold	Metric	Bootstrap Mean	95% CI Lower	95% CI Upper
**0.11**	**Sensitivity**	**90.7%**	**84.5%**	**95.2%**
	Specificity	36.9%	29.1%	52.4%
	Precision	25.5%	21.4%	30.4%
	Workload Reduction	31.6%	25.2%	45.5%
**0.15**	Sensitivity	84.4%	77.9%	90.9%
	Specificity	49.5%	33.3%	56.1%
	Precision	28.4%	24.0%	33.1%
	Workload Reduction	43.1%	28.3%	49.3%
**0.18**	Sensitivity	80.7%	70.9%	87.6%
	Specificity	54.5%	47.7%	63.2%
	Precision	29.6%	25.6%	34.0%
	Workload Reduction	47.8%	41.2%	56.5%
**0.22**	Sensitivity	73.0%	60.0%	82.9%
	Specificity	62.2%	53.4%	69.0%
	Precision	31.4%	26.7%	36.2%
	Workload Reduction	55.5%	45.8%	62.9%

To assess model generalizability and avoid overfitting, we performed a 5-fold stratified cross-validation. The model demonstrated consistent performance across all five folds, with a mean AUC of 0.723 (SD: 0.041). At the 0.11

© 2025 .Liu S et al. *JAMA Network Open*.

threshold, performance was also stable, with a mean sensitivity of 89.1% (SD: 0.067). These results support the model's ability to generalize to new data.

Several limitations of this approach should be considered. The model relies on a single predictor (the LLM's usefulness score), assumes a linear relationship between this score and the log-odds of the outcome, and does not account for potential clustering of suggestions within the same order set. Its performance may also vary if suggestion patterns or expert rating behaviors change over time.

In conclusion, the logistic regression filter demonstrates a statistically significant and stable ability to discriminate between useful and non-useful suggestions. The systematic threshold selection process, comprehensive diagnostics, and rigorous uncertainty quantification provide strong evidence for the model's validity. The selected threshold of 0.11 achieves an effective and well-characterized balance between preserving valuable clinical suggestions and reducing physician review burden.

eAppendix 5. Proposed Framework for Quantitatively Evaluating the Evidence Traceability of Generated Suggestions.

We propose a multi-phase framework to quantitatively measure how well suggestions are grounded in the provided evidence, combining both human expert evaluation and automated methods.

Phase 1: Enhancing the System for Traceability

The first step would be to modify the multi-agent system's output. For every suggestion generated, the system would be required to also output the specific text snippet(s) from the source documents (e.g., the sentence from a PubMed abstract or a guideline) that it used as the primary basis for that suggestion. This creates an explicit (Suggestion, Evidence) pair for evaluation.

Phase 2: Designing Quantitative Metrics

With these pairs, we could then deploy the following quantitative evaluations:

1) Human-in-the-Loop Evaluation

A panel of physician experts would review a random sample of the (Suggestion, Evidence) pairs. They would be asked to answer the following questions to generate quantitative metrics:

- Metric 1: Evidence Traceability (Binary): "Does the provided evidence snippet directly support the clinical claim made in the suggestion?" (Yes/No).
  Output: Traceability Rate. The percentage of suggestions for which the answer is "Yes." This is the most direct measure of the system's "truthfulness."

- Metric 2: Faithfulness Score (Likert Scale): "On a scale of 1-5, how faithful is the suggestion to the provided evidence?"
  1 = Contradiction: The suggestion misrepresents or contradicts the evidence.
  3 = Related but Not Supported: The suggestion is on the same topic but is not directly derivable from the evidence snippet.
  5 = Directly and Accurately Supported: The suggestion is a direct, accurate, and logical conclusion from the evidence.
  Output: Mean/Median Faithfulness Score. This provides a more nuanced measure of grounding quality than a simple binary metric.

- Metric 3: Evidence Sufficiency (Binary): "Is the provided evidence snippet, on its own, *sufficient* to justify making this clinical suggestion?" (Yes/No).

Output: Sufficiency Rate. This metric would identify instances of "over-reaching," where the LLM might take a minor or out-of-context statement and generate an overly strong suggestion.

2) Automated Evaluation (For Scalability)

Because expert human review is resource-intensive, we would also develop and validate automated metrics against our human-rated gold standard.

- Metric 4: Semantic Similarity Score: We would calculate the cosine similarity between the sentence-embedding vectors of the suggestion and its cited evidence.
  Output: Mean Semantic Similarity. While not a perfect proxy for factual consistency, a consistently low score could indicate a systemic failure to align suggestions with sources.

- Metric 5: Natural Language Inference (NLI) Score: We would frame the task as an NLI problem, where the "premise" is the evidence snippet and the "hypothesis" is the suggestion. A pre-trained NLI model and LLM-as-a-judge would classify the relationship as *Entailment*, *Contradiction*, or *Neutral*.
  Output: Entailment Rate. The percentage of pairs classified as "Entailment" would serve as an automated proxy for traceability.

Implementation and Impact on System Improvement

By implementing this framework, we could directly diagnose weaknesses in the generation process. For example, if we found a high Traceability Rate but a low Sufficiency Rate, it would imply the agent is good at finding relevant facts but poor at judging their clinical weight. These fine-grained, quantitative metrics would serve as a much better guide for targeted prompt engineering and system refinement than the current, more general “usefulness” rating.

© 2025 .Liu S et al. *JAMA Network Open*.
