# Supplementary material for: Optimizing Order Sets With a Large Language Model–Powered Multiagent System
Source: JAMA Netw Open. 2025 Sep 23;8(9):e2533277. doi: 10.1001/jamanetworkopen.2025.33277 (PMC12457977; doi:10.1001/jamanetworkopen.2025.33277)
Supplement: Supplement 2. — Data Sharing Statement [file jamanetwopen-e2533277-s002.pdf]

Data Sharing Statement

Liu. Optimizing Order Sets With a Large Language Model–Powered Multiagent System. *JAMA Netw Open*. Published September 23, 2025. doi:10.1001/jamanetworkopen.2025.33277

Data
Data available: No

Additional Information
Explanation for why data not available: This study uses order sets from Vanderbilt University Medical Center that are published online in the Epic Community Library. Researchers with access to the Epic system can retrieve this data source.
